# Speech Development Across Subgroups of Autistic Children: A Longitudinal Study

**DOI:** 10.1007/s10803-022-05561-8

**Published:** 2022-04-19

**Authors:** Kate Broome, Patricia McCabe, Kimberley Docking, Maree Doble, Bronwyn Carrigg

**Affiliations:** 1 Present Address: Speech Pathology, Sydney School of Health Sciences, Faculty of Medicine and Health, The University of Sydney, Susan Wakil Health Building D18, Western Avenue, Sydney, NSW 2006 Australia; 2 South West Sydney Local Health District, Sydney, Australia; 3 Present Address: Perth Children’s Hospital, Perth, Australia; 4 Curtin University, Perth, Australia; 5 Speech Pathology, Sydney Children’s Hospital, Randwick, Sydney, Australia

**Keywords:** Speech, Autism, Child, Longitudinal

## Abstract

Subgroups of children with different speech profiles have been described however, little is known about the trajectories of speech development or stability of subgroups over time. This longitudinal study described both speech trajectories and subgroup stability of 22 autistic children, aged 2;0–6;11 years, over 12 months. Independent and relational speech analyses, vocabulary size and nonverbal communication were used in clustering. Results suggest varied speech trajectories, particularly for children with ‘low language and low speech’ at Time 1. Receptive vocabulary and consonant inventory at Time 1 may predict speech outcomes after 12 months. A small subgroup of children (n = 3) present with low expressive vocabulary and speech but higher receptive vocabulary and use of gestures. This unique profile remained stable.

**Supplementary Information:**

The online version contains supplementary material available at 10.1007/s10803-022-05561-8.

Autistic children form a heterogeneous neurodiverse population. A diagnosis of autism is generally characterized by the presence of specific restricted and repetitive interests and behaviors, in addition to differences in social communication abilities compared with non-autistic children (DSM-5; American Psychiatric Association, 2013). Although not a core feature, many children on the spectrum are also diagnosed with language impairments (Levy et al., 2010) and it follows that communication research with autistic children has largely focused on the areas of social communication and language. The speech capacity of these children has, until recently, been largely overlooked. The small body of research examining the speech of autistic children outlines a few key findings: (1) prelinguistic children may produce fewer consonants and less canonical babbling than neurotypical children (Paul et al., 2011; Plumb & Wetherby, 2013; Schoen et al., 2011); (2) highly verbal children present with higher rates of delayed or disordered speech (Cleland et al., 2010; Shriberg et al., 2001, 2011); and (3) a small subgroup of minimally verbal children may have a significant co-occurring speech sound disorder (SSD; Broome et al., 2021; Chenausky et al, 2019; Rapin et al, 2009).

Despite this growing body of literature, little is known about the development of speech in autistic children. Few longitudinal studies of the communication development of autistic children have included speech variables. Those that have, focus on the early vocalizations and consonant inventories of minimally verbal children as important predictors of later expressive language ability (see McDaniel et al., 2018 for review). The definition of ‘minimally verbal’ varies depending on the study, but generally refers to children using fewer than 20 words and not yet at phrase level expressive language (Chenausky et al., 2019; Thurm et al., 2015; Yoder et al., 2015). Given that a child’s functional expressive language ability is associated with fewer maladaptive behaviors (Dominick et al., 2007; Hartley et al., 2008) and better social outcomes (Billstedt et al., 2005; Howlin et al., 2000), identifying predictors of expressive language outcomes is important. In the most recent study of this kind, Saul and Norbury (2020) aimed to expand previous findings by Yoder et al. (2015) that parental responsiveness, child response to joint attention, child communicative intent and consonant inventory were unique predictors of expressive language growth. Saul and Norbury (2020) studied the expressive language development of 27 minimally verbal autistic children, aged 2–5 years, over 12 months and used the same predictors as Yoder et al. but with a more expanded measure of phonetic repertoire. Consonant inventory and phonetic repertoire were found to be significant predictors of expressive language growth. These results highlight the importance of a child’s early speech capacity to later expressive language ability although add little information regarding a child’s speech progression.

In neurotypical development, early speech follows a predictable pattern of development from early cooing sounds to simple and then more complex babbling forms (Nathani et al., 2006; Tager-Flusberg et al., 2009). The sounds in a child’s canonical babbling are similar to those in their first words, suggesting that early vocalizations strongly correlate with later expressive language development (McCune & Vihman, 2001; McGillion et al., 2017; Oller, 2000; Watt et al., 2006). As the child grows and develops, so too does their speech. By the time a neurotypically developing child is 5 years old they can produce most consonants and vowels and their speech can be understood by familiar and unfamiliar people (Flipsen, 2006; Hustad et al., 2021; McLeod & Baker, 2017). The final stage towards adult-like speech is to refine their production of polysyllable words, their acquisition of the later developing consonants (i.e. ‘th’) and small adjustments to their expressive prosody. For autistic children with more advanced speech and language levels, it could be hypothesized that their speech development would follow this pattern. It remains unclear if the same is true for autistic children with suspected co-occurring speech sound disorders.

There is a paucity of available literature detailing the speech development of autistic children. Different subgroups of children based on their speech capacity are beginning to emerge in the literature but, to date, we know little about the trajectory of speech development for the different subgroups (Broome et al., 2021; Chenausky et al., 2019; Rapin et al., 2009). All three published studies report a subgroup of children with average speech abilities, a subgroup of children with very low speech and language, and one or more subgroups of children with suspected speech sound disorders (SSDs). Specifically in relation to the SSD subgroups, Rapin et al. (2009) reported a subgroup of four (6%) children, aged 7–9 years, with ‘profoundly’ impaired phonology, stronger receptive language, and average nonverbal IQ. Chenausky et al. (2019) described two SSD subgroups in a cohort of ‘minimally verbal’ and ‘low verbal’ children, aged 4;4–18;10 years: (1) a subgroup of 13 (24%) children with suspected of having Childhood Apraxia of Speech (CAS), and (2) a subgroup of 16 (30%) children described as having non-CAS speech difficulties. Finally, Broome et al. (2021) described a subgroup of 3 (13%) children, aged 2;0–6;11 years, with low speech and expressive vocabulary, but higher receptive language and use of gestures. Given the methodological differences between studies, it remains unclear if the subgroups of children with suspected SSDs had similar presentations. Without prospective longitudinal speech studies with autistic children, little is known about their speech development either individually or in the different subgroups. It is also unknown whether the emergent subgroups remain stable over time or if some children’s speech progresses on a different trajectory to other group members.

Defining patterns of speech development is important to further our understanding of the different speech profiles of autistic children and the speech outcomes for these children. Identifying the barriers to communication for children informs diagnosis and guides intervention. Some autistic children may present with a co-occurring SSD which impacts their ability to develop intelligible speech and this may require targeted intervention, as it does with non-autistic children. The current prospective longitudinal study aimed to: (1) examine the stability of speech subgroups over 12 months, and (2) describe which variables may explain changes in speech capacity over time.

## Method

This study used a prospective longitudinal descriptive design to evaluate the speech development of autistic children. The research protocol was approved by the Human Research Ethics Committee of The University of Sydney (2012/712) and (2012/1305) and written consent was obtained from parents on behalf of all participants.

### Participants

As described in Broome et al (2021), participants were recruited from an autism early intervention service provider, ASPECT, in the greater metropolitan area of Sydney, and from private speech pathologists in the Sydney area who either listed ASD as an area of interest on the Speech Pathology Australia website or a member of the ASD evidence-based practice interest group in Sydney, Australia. Parents interested in participating contacted the first author initially and were screened for eligibility over the telephone. Standard questions regarding the following inclusion and exclusion criteria were asked.

Children were excluded from the study if they were: (a) born at less than 36 weeks gestational age, (b) diagnosed with co-occurring developmental disorders or genetic syndromes, or (c) had any uncorrected hearing or visual deficits. All participants completed an oral motor screen with the first author at the initial assessment. Children were excluded from the study if there was asymmetry or weakness of the oral musculature, resulting in significant drooling and/or dysarthria. Children were also excluded if there were any oral structural abnormalities (e.g., cleft palate). Three children were excluded from participating in the study as they were diagnosed with co-occurring developmental disorders (i.e., cerebral palsy).

A total of 22 children participated (20 males and 2 females) who at entry to the study: (a) were aged between 2;0–6;11 years, (b) had a documented diagnosis of ASD in accordance with the Diagnostic and Statistical Manual of Mental Disorders—Fourth Edition Text Revision (American Psychiatric Association, 2000) or Fifth Edition (American Psychiatric Association, 2013), and (c) had a developmental or cognitive assessment or the intention to complete a developmental assessment within the time frame of the broader longitudinal study. All participants were reported to be using speech-like vocalizations and had English as their primary language. A description of participants is provided in Table 1. Twenty-three participants were included at Time 1. Only one participant was unavailable for follow-up assessment at Time 2, leaving 22 participants in the longitudinal study.

**Table 1:** Description of participants

Variable	n	*M*	*SD*	Range
Time 1 age (months)	22	46	14.9	24–74
Time 2 age (months)	22	58.4	15	37–86
CSBS				
Time 1 CC	9	54.6	8.3	47–70
Time 2 CC	6	51.5	6.3	47–64
PLS-4				
Time 1 AC	14	66.1	14.9	50–92
Time 2 AC	16	72.7	16.7	50–101
Time 1 EC	14	65.6	14.2	50–85
Time 2 EC	16	71.3	14.6	50–92
GMDS-ER				
Nonverbal DQ	13	59.3	23.4	21.3–108.3
Stanford-binet				
Nonverbal IQ	3	88	14.5	74–103
WPPSI-III				
Nonverbal IQ	3	99	20.7	82–122
WISC-V				
Nonverbal IQ	1	86		

*CSBS CC* communication composite sum of scaled scores based on level of functioning, *PLS-4* Preschool language scale fourth edition standard scores, *AC* auditory comprehension, *EC* expressive communication, *TLS* total language score, *GMDS-ER* griffiths mental development scale—extended revised, *DQ* developmental quotient, *IQ* intelligence quotient, *WPPSI-III* Wechsler Preschool and primary scale of intelligence third edition, *WISC-V* Wechsler intelligence scale for children fifth edition

The current study aimed to describe the optimal speech capacity of a heterogenous cohort of autistic children. Considering the lengthy assessment battery and the professional skills of the authors, completing developmental or cognitive assessment was outside the scope of this study. Instead, families provided results from previous developmental or cognitive assessments. Given the varied assessments, reporting standards, and likely differences in assessment methodology, this data was unable to be used in the statistical approaches in this study. Instead, these scores provide further descriptive data of the participants in this study and highlight the heterogeneity in our cohort. The results from a formal developmental or cognitive assessment were not available for two participants. Available developmental scores on the Griffiths mental developmental scales—extended revision (GMDS-ER; Luiz et al., 2004) were available for 13 participants and reported as a developmental quotient (DQ) as many participants scored below the 1st percentile. The DQ was calculated by dividing age equivalent by chronological age (CA) multiplied by 100. A nonverbal developmental quotient (NVDQ) was calculated from the performance scale and the verbal developmental quotient (VDQ) was calculated from the hearing and speech scale. The results of a cognitive assessment were available for seven participants. For these children, the verbal intelligence quotient (VIQ) and nonverbal intelligence quotient (NVIQ) are presented.

### Assessment Measures

Participants completed a comprehensive communication assessment battery, including direct language and speech measures, spontaneous speech sampling, and parent questionnaires. Assessment measures were completed at both Time 1 and at Time 2, 12 months later and are detailed in Table 2. All assessment sessions were video- and audio-recorded. To capture each participant’s optimal communication ability, most assessments (95%) were completed over two sessions at the participants’ homes. One child was assessed at the on-campus clinic at The University of Sydney (5%). Every effort was made to complete the primary assessment battery with all participants, however if a child was unable to engage with the assessment or reach basal level on an individual assessment an alternative assessment was presented. To develop our understanding of the capacity of these children, participants were not excluded from this study if they were unable to complete one or more primary assessments. Parents were also asked to record the details of the child’s early intervention, including hours and details per week.

**Table 2:** Assessments

Variable	Primary assessment	Secondary assessment
Speech		
Single-word naming	SWPT^a^	FWFST
Speech sample	Spontaneous speech sample	–
Language		
Parent questionnaire	CDI	–
Standardized assessment	PLS-4	CSBS

^a^*POP* Polysyllable preschool test (Baker, 2013) at Time 1. *CDI* MacArthur-Bates communicative development inventory—words and gestures (Fenson et al., 2007), *CSBS* Communication and symbolic behavior scales—behavior sample (Wetherby & Prizant, 2002), *FWFST* First words first sentences test (Gillham et al., 1997), *PLS-4* Preschool language scale—fourth edition (Zimmerman et al., 2002), *SWPT* single word polysyllable test (Gozzard et al., 2013)

#### Capturing Language Ability

The Preschool language scales—fourth edition (PLS-4; Zimmerman et al., 2002) was presented to all participants. The PLS-4 is a standardized language assessment for children from birth to 6;11 years. One participant was 7;2 years at Time 2, when this tool was readministered. While this age is out of the range for the PLS-4, this participant performed at very low language levels, making the PLS-4 an appropriate assessment tool. This participant scored a standardized score of 50 (1%ile) for all scores at Time 1. His Time 2 scores were compared to children between 6;6–6;11 and again he scored 50 (1%ile) for all standard scores. It is assumed that this score reflects his performance. Sixteen participants completed the PLS-4 at Time 2 and comparative data across time points was collected for 14 participants.

Where it was not possible to obtain a basal level on the PLS-4, the Communication and symbolic behavior scales—behavior sample (CSBS; Wetherby & Prizant, 2002) was completed. The CSBS is a standardized assessment that assesses language comprehension and word use, in addition to other important aspects of very early communication development, such as social-affective signaling, nonverbal communication and joint attention. As children were older than the normed sample, scores reported are based on the child’s language stage as recommended in the manual (Wetherby & Prizant, 2002, p. 61).

A parent questionnaire was used to ensure a consistent measure could be used with all participants. The MacArthur-Bates communicative development inventory—words and gestures (CDI; Fenson et al., 2007) was completed by all parents at Time 1 and 2. The CDI is a 396-word checklist of a child’s receptive and spoken expressive vocabulary, in addition to the use of 18 early gestures (i.e., communicative and games/routines) and 45 later emerging gestures (i.e., actions with objects, pretending to be a parent, imitating adult actions). The Words and Gestures form provides standard scores for children aged 0;8–1;4 years. For the purposes of this study the form was used to tally the participant’s vocabularies and only raw data is reported. Parents separately marked their child’s words ‘understood’ and words ‘says’ instead of ‘words understood’ and ‘words understood and says’ as guided on the form. This allowed for separate measures of spoken expressive and receptive vocabulary. The CDI has been used by several research groups as a measure of vocabulary in autistic children (e.g., Charman et al., 2003; Luyster et al., 2007; Stone & Yoder, 2001). Like these authors, we required one instrument that could provide data on all children in our study. We also wanted a measure of nonverbal communication that could be used with all participants.

#### Capturing Speech Capacity

A single-word naming task was presented to all participants. Ideally, the assessment tool would assess all phonemes in all word positions, in addition to a polysyllabic word assessment (Broome et al., 2017). However, to reduce the length of the assessment battery and increase the likelihood of completing the entire battery, only a polysyllabic assessment was included. A child’s ability to produce polysyllable words provides phonological and stress pattern data that may not be apparent from spontaneous speech samples in which a child may choose to use simpler word shapes. At Time 1, participants were presented with the Toddler polysyllable test—second edition (POP: Baker, 2013), a 20-word task. At Time 2, the Single Word Polysyllable Test (SWPT: Gozzard et al., 2004) was used to expand this data. The POP and SWPT are similar single-word naming tasks. The SWPT is a 50-word measure, including 19 of the 20 words included in the POP. The additional 31 words in the SWPT present similar word shapes and phonological complexity to the POP. At Time 2, 10 participants were able to complete the SWPT.

Participants unable to complete the polysyllabic word assessment were presented with the First Words First Sentences Test (FWFST: Gillham et al., 1997). This single-word naming task presents early developing vocabulary as photographs rather than symbolic pictures, making it easier for children at earlier levels of linguistic development to complete. Nine participants completed the FWFST at Time 2. Although the FWFST does not present children with the same complexity of word shapes as the SWPT and POP, children who completed this task were not using complex polysyllable words in their spontaneous speech. Their performance on the FWFST likely represented their optimal speech capacity.

A spontaneous sample of speech and speech-like utterances was also collected for all participants. Speech-like utterances included babble, jargon and echolalia. Echolalia was included in the spontaneous speech samples as it demonstrated a participant’s speech capacity. For children who were verbal, a minimum of 50 utterances were collected during parent–child play lasting at least 10 min. If children did not produce many utterances during play with a parent, the spontaneous speech sample was taken from the CSBS recording. Three participants were at a prelinguistic level, defined as producing less than 10 recognizable words (Broome et al., 2017; Stoel-Gammon, 1989) at Time 2. Vocalizations produced concurrently with background noise, such as an adult talking or dog barking, were excluded from the sample. Utterances were categorized as babble if a target word was unable to be identified after watching the recording three times.

### Data Preparation

Broad phonemic transcription was completed on all single-word naming task responses and entered into Phon 3.1 Computer Software (Hedlund & Rose, 2020). Independent and relational speech analyses were completed.

#### Independent Speech Analyses

##### Consonant Inventories

The total number of consonants for each participant was tallied from the single-word naming task and spontaneous speech sample. For prelinguistic participants, the number of consonants was calculated from the entire assessment battery. Consonants were categorized as Early 8, Middle 8 or Late 8 (Shriberg, 1993).

##### Syllable Shapes

Responses on the single-word naming task were analyzed according to syllable shapes. Syllables were those containing a nuclei vowel (V) and possibly one or more pre- or post-vocalic consonants (C). Consonant blends were represented by the number of consonants (C) within the syllable shape. For example, VCC would indicate a syllable with a vowel and post-vocalic consonant blend of two consonants (e.g. ‘ink’). The number of different syllable shapes is reported.

#### Relational Speech Analyses

##### Phoneme Accuracy

Percent consonants correct (PCC), percent vowels correct (PVC), and percent phonemes correct (PPC; Shriberg & Kwiatkowski, 1982) were calculated from the single-word naming task completed by the 19 verbal participants using Phon 3.1 Computer Software (Hedlund & Rose, 2020).

### Reliability

The first author completed broad phonemic transcription for all responses on the single-word naming task and then re-transcribed 23% of the data to check for intra-rater reliability. An independent researcher transcribed 23% of the single-word naming tasks, randomly selected using random.org. Intra-rater reliability was 96.8% and inter-rater reliability was 93.1%.

The first author tallied the total number of different consonants from the entire assessment battery. The first author completed these ratings again for 23% of participants more than 6 months after the initial analysis. An independent postgraduate SLP tallied the total number of consonants for five (23%) participants. Intra-rater reliability was 98.6% and inter-rater reliability was 95.3%.

### Data Analysis

**Fig. 1:**
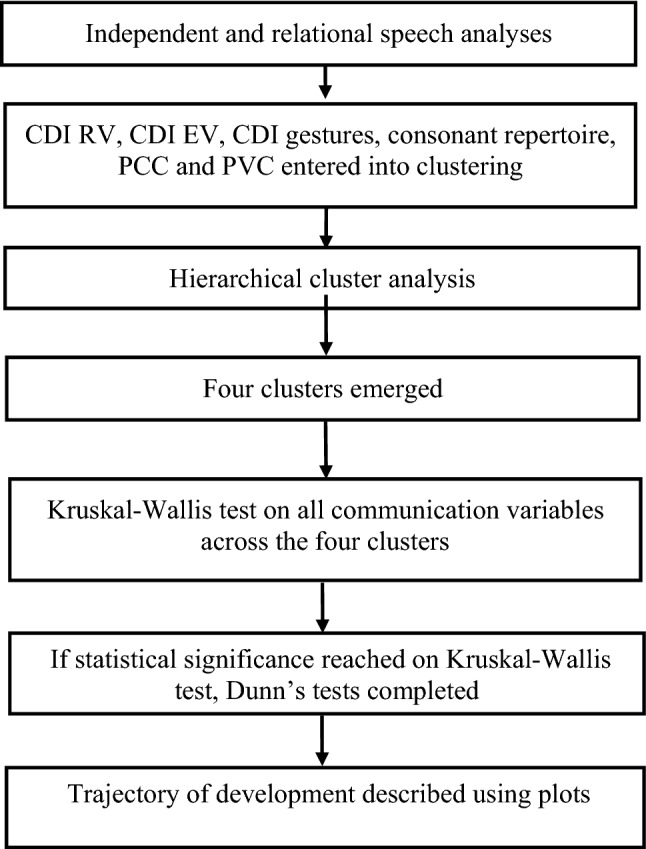
Process of data analysis. *CDI RV* number of words understood on CDI (Fenson et al., 2007), *CDI EV* number of words expressed on CDI (Fenson et al., 2007), *PCC* percent consonants correct, *PVC* percent vowels correct

The primary analysis conducted in this research was a hierarchical cluster analysis (HCA). The process of analysis, from descriptive data to HCA and then to describing subgroups of children is outlined in Fig. 1.

### Hierarchical Cluster Analysis

Agglomerative hierarchical cluster analysis with Euclidean distance (Hastie et al., 2009) was used to explore whether homogeneous subgroups exist within the cohort. In this paper, Time 2 data are analyzed in order to examine stability of subgroups from Time 1 to Time 2. The data derived from clustering is visually presented on a dendrogram, a tree-based representation of the participants. In agglomerative clustering, the dendrogram is built bottom-up. At the bottom of the dendrogram, each participant is initially in their own cluster. Participants join together hierarchically, first joining with those participants most similar, and eventually to the participants most dissimilar. The dissimilarity measure, of which Euclidean is the most common, determines the similarity of two individual participants (James, 2013). Participants most similar join at a low height on the dendrogram. A measure of dissimilarity between sets of data is needed to determine how clusters combine. This is referred to as linkage. In this study, complete linkage was used. Complete linkage, also known as furthest neighbor, defines the difference between two groups of participants as the distance between the two most dissimilar participants in those groups. Participants who merge higher in the dendrogram are less similar than those who fuse at a lower height.

Six Time 2 communication measures were used as clustering variables and entered into R (R Core Team, 2017). These included the CDI receptive vocabulary, CDI expressive vocabulary, CDI number of gestures, consonant repertoire, PCC and PVC. These variables described all aspects of a participant’s communication ability, including language, nonverbal communication and independent and dependent speech measures were selected. As variables in this study are measured on different scales, Time 2 data was converted into z scores prior to clustering. This method was used previously to report on Time 1 data (Broome et al., 2021).

The number of clusters is determined by drawing a horizontal line across the dendrogram. Determining the most appropriate level to cut the dendrogram does in part require the researchers to ascertain which solution may best suit the data. For some dendrograms, researchers may explore more than one solution. Once a solution is decided upon, then it can be statistically evaluated to determine if differences between clusters on communication variables reached statistical significance. This was done for all variables in this study through a series of Kruskal–Wallis tests with alpha was set at 0.05. Due to the exploratory nature of this study and given the small n, using a stricter alpha level may result in higher type II errors. Variables that were statistically different on the Kruskal–Wallis test were then subjected to Dunn’s test across clusters. Dunn’s test analysis examined which clusters differed on which variables. This process is outlined in Fig. 1.

### Trajectories of Speech Development

Plots were used to visualize the communication profiles of the Time 2 clusters and to illustrate change in speech over 12 months. To plot variables measured on different scales and to visualize change over time, data from Time 1 and Time 2 were converted into z scores collectively. The mean z scores at Time 1 and Time 2 were displayed on separate plots. Comparing these two plots illustrates change over 12 months across the six communication variables for the Time 2 clusters.

## Results

### Describing the Clusters

**Fig. 2:**
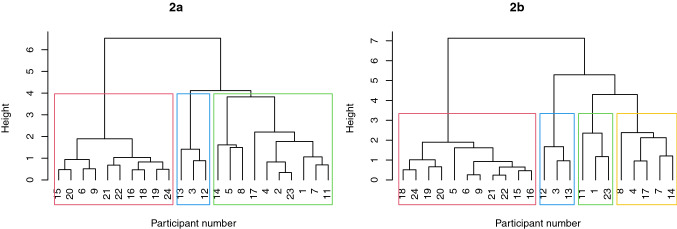
**a** Time 1 dendrogram (Broome et al., 2021; reprinted with permission from ASHA). **b** Time 2 dendrogram

Agglomerative hierarchical cluster analysis was used to explore whether homogeneous subgroups exist within the cohort based on Time 2 data. Euclidean distance with complete linkage was used. By comparing dendrograms from Time 1 and Time 2 (Fig. 2) the stability of cluster membership over 12 months can be analyzed. The dendrogram from Time 1 clustering is included for comparison (with permission from JSLHR). This dendrogram produced a 3-cluster solution (Fig. 2a).

The Time 2 dendrogram (Fig. 2b) illustrates a 4-cluster solution, by horizontally cutting the dendrogram at height 3. Three clusters emerge if you cut the dendrogram higher, with the children in Cluster C and D fusing. Merging high in the dendrogram suggests a less homogeneous subgroup (James, 2013) and so was not explored further.

Parents were asked to indicate how many hours their child spent in speech pathology and other early intervention services on a weekly basis. As participants were recruited from an autism early intervention provider or from private speech pathologists, all children in this study attended at least weekly early intervention. Given the nature of the transdisciplinary model used in these services, not all parents were clear on which service their child was receiving at any time. Most participants had weekly speech pathology, although some parents were unable to indicate if this included spoken language, AAC, or speech intervention. Hours of speech pathology or early intervention were not analyzed statistically, as it was decided that the information may be misrepresentative and misleading.

Kruskal Wallis tests were completed to statistically analyze if the four clusters differed on communication variables and age. The results of these tests are presented in Table 3. The PLS-4 Auditory Comprehension scores, proportion of CV syllables and proportion of 3-consonant blends did not reach statistical significance on the Kruskal Wallis test and will not be explored further. It is important to recall that only 16 participants completed the PLS-4 at Time 2. Further, no child in Cluster B or C produced three-consonant blends. Dunn’s tests were performed for the remaining communication variables and age to ascertain exactly which clusters differed on which variables. The results from the Dunn’s tests are shown in Table 4. The characteristics of each cluster are described below.

**Table 3:** Characteristics of clusters

Variable	Cluster A			Cluster B			Cluster C			Cluster D			χ^2^	*p*
	n	Mean (SD)	Range	n	Mean (SD)	Range	n	Mean (SD)	Range	n	Mean (SD)	Range		
T2 Age	11	64.9 (11.8)	46–80	3	70.7 (18.6)	50–86	3	44 (4.4)	39–47	5	45.2 (7.2)	37–55	11.1	.011*
CDI RV	11	379.9 (20.6)	328–396	3	332.7 (54)	289–393	3	94 (74.6)	13–160	5	169.4 (73.4)	60–244	16	.001*
CDI EV	11	363.3 (48.7)	230–396	3	32.7 (35.5)	6–73	3	3 (3)	0–6	5	103 (68.6)	35–191	17.6	.0005*
Gestures	11	54.5 (6.6)	45–63	3	49 (7.9)	40–55	3	29.7 (8)	22–38	5	42.6 (7.2)	35–52	11.5	.009*
PLS AC	11	77.6 (14.9)	50–101	3	55.7 (9.8)	50–67				2	70.5 (24.7)	53–88	4.2	.12
PLS EC	11	76.4 (12.3)	50–92	3	50 (0)					2	75.5 (2.1)	74–77	6.1	.047*
Consonants	11	21.4 (1.5)	19–24	3	7.3 (1.2)	6–8	3	3.3 (2.5)	1–6	5	15.4 (3.4)	13–21	17.1	.0007*
Early 8	11	8 (0)		3	4.7 (2.1)	3–7	3	2.6 (2.5)	0–5	5	7.8 (0.4)	7–8	18.4	.0004*
Middle 8	11	7.7 (0.5)	7–8	3	1.7 (1.2)	1–3	3	0.3 (0.6)	0–1	5	4.4 (1.7)	3–7	18.2	.0004*
Late 8	11	5.6 (1.1)	4–8	3	1 (1)	0–2	3	0.3 (0.6)	0–1	5	3.2 (1.6)	2–6	15.1	.001*
Syllable shape														
V	11	4.9 (1.5)	2.9–7.9	3	49.5 (30.7)	14.6–72.5	3	62.5 (22)	43.2–86.5	5	9.7 (4.6)	4.7–15.8	15.3	.001*
CV	11	57 (11.4)	22.9–61.8	3	45.6 (26.1)	27.5–75.6	3	37.2 (21.9)	13.5–56.8	5	52.9 (16.8)	31.6–72.5	3.4	.33
CVC	11	21.5 (6.2)	17.2–40	3	4.8 (4.9)	0–9.8	3	0.3 (0.5)	0–0.9	5	25 (10.7)	10.1–34.2	12.7	.005*
VC	11	3.9 (1.2)	2–5.7	3	0		3	0		5	6 (5.6)	2.2–15.8	12.8	.005*
CCV	11	6.4 (2.5)	0–9.8	3	0		3	0		5	2.2 (2.1)	0–4.7	14.1	.003*
CCVC	11	2.4 (3)	0.7–11.4	3	0		3	0		5	2.5 (2.6)	0–6.3	10.1	.018*
CVCC	11	3.6 (3.6)	2.5–14.3	3	0		3	0		5	1.3 (1.8)	0–3.4	10.6	.014*
CCVCC	11	0.3 (0.9)	0–2.9	3	0		3	0		5	0.2 (0.5)	0–1.1	1.1	.77
CVCCC	11	0		3	0		3	0		5	1.1		3.4	.33
PCC	11	84.9 (8.7)	71.4–95.3	3	13.3 (9.3)	2.7–19.7				5	54.9 (17.2)	42.9–84.2	12.3	.002*
PVC	11	94.4 (3.7)	88.2–99.4	3	22.3 (3.4)	18.9–25.6				5	82.4 (13.2)	68.7–100	9.7	.008*
PPC	11	89 (6.4)	78.6–96.8	3	16.6 (6.3)	9.7–21.9				5	65 (15.1)	53.2–90.2	12.3	.002*

*p ≤ .05 Kruskal–Wallis test

*T2 Age* age in months at Time 2, *CDI RV* Number of words understood on CDI (Fenson et al., 2007), *CDI EV* number of words expressed on CDI (Fenson et al., 2007), *Gestures* number of gestures used on CDI (Fenson et al., 2007), *PLS AC* Preschool language scale—fourth edition auditory comprehension score, *PLS EC* Preschool language scale—fourth edition expressive communication score, *Consonants* total number of consonants in phoneme repertoire, Early 8, Middle 8, Late 8 consonants (Shriberg, 1993), *Syllable shapes* proportion of syllable shapes, *V* vowel, *C* consonant, *PCC* percent consonants correct, *PVC* percent vowels correct, *PPC* percent phonemes correct

**Table 4:** Multiple pairwise comparisons using Dunn’s test

Variable	Cluster A and B		Cluster A and C		Cluster A and D		Cluster B and C		Cluster B and D		Cluster C and D	
	z	*p*	z	*p*	*z*	*p*	*z*	*p*	*z*	*p*	*z*	*p*
T2 Age	− 0.52	.3	2.3	.011*	2.5	.007*	2.2	.013*	2.3	.011*	− 0.23	.41
CDI RV	1	.16	3.2	.0007*	3.2	.0008*	1.7	.041*	1.4	.076	− 0.5	.31
CDI EV	2.6	.004*	3.5	.0002*	2.5	.007*	0.7	.24	− 0.5	.29	− 1.3	.09
Gestures	0.8	.2	3	.0012*	2.2	.013*	1.8	.039*	0.9	.18	− 1.1	.14
PLS EC	2.5	.007*			0.4	.36			− 1.5	.073		
Consonants	2.8	.002*	3.5	.0003*	2	.022*	0.5	.31	− 1	.15	− 1.6	.05
Early 8	3.1	.001*	3.5	.0002*	0.6	.28	0.3	.37	− 2.3	.011*	− 2.7	.004*
Middle 8	2.9	.002*	3.5	.0002*	2.3	.011*	0.5	.3	− 0.9	.19	− 1.5	.07
Late 8	2.9	.002*	3.3	.0006*	1.8	.036*	0.3	.37	− 1.2	.11	− 1.6	.06
Syllables												
V	− 2.9	.002*	− 3.1	.001*	− 1.8	.036*	− 0.1	.45	1.3	.099	1.4	.077
CVC	2.3	.01*	2.7	.003*	− 0.3	.39	0.3	.38	− 2.3	.011*	− 2.6	.004*
VC	2.6	.005*	2.6	.005*	− 0.1	.47	0	0.5	− 2.4	.009*	− 2.4	.009*
CCV	2.8	.003*	2.8	.003*	2.3	.01*	0	0.5	− 0.8	.22	− 0.8	.22
CCVC	2.4	.009*	2.4	.009*	0.1	.45	0	0.5	− 2	.022*	− 2	.022*
CVCC	2.5	.006*	2.5	.006*	1.2	.12	0	0.5	− 1.4	.08	− 1.4	.08
PCC	3.2	.0007*			2.3	.012*			− 1.2	.12		
PVC	3	.001*			1.6	.057			− 1.5	.067		
PPC	3.2	.0007*			2.3	.012*			− 1.2	.12		

*p ≤ .05 Dunn’s test

*CDI RV* number of words understood on CDI (Fenson et al., 2007), *CDI EV* number of words expressed on CDI (Fenson et al., 2007), *Gestures* number of gestures used on CDI (Fenson et al., 2007), *Consonants* total number of consonants in phoneme repertoire; Early 8, Middle 8, Late 8 consonants (Shriberg, 1993), *Syllable* proportion of syllable shapes in spontaneous speech sample, *V* vowel, *C* consonant, *PCC* percent consonants correct, *PVC* percent vowels correct, *PPC* percent phonemes correct

### Cluster A: High Receptive, High Expressive, High Gestures, High Speech

Children in Cluster A presented with high language, use of gestures and speech capacity. The mean age of the 11 children in Cluster A was 64.9 (*SD* 11.8) months, not statistically different to the children in Cluster B, but older than those in Cluster C and D. The mean NVIQ of the seven Cluster A children with available data was 92.4 (*SD* 15.8, range 74–122) and the mean NVDQ of the remaining four children was 70.6 (*SD* 18, range 43.8–81.4). It is important to note, that comparing cognitive scores from different tests is not without limitation and some caution is needed. Pairwise comparisons using Dunn’s test indicated that Cluster A children presented with statistically higher spoken expressive vocabularies than children in any other cluster. Interestingly, their Expressive Communication scores on the PLS-4 did not differ significantly from Cluster D, although only two participants in Cluster D completed the PLS-4 at Time 2. This may indicate that two participants in Cluster D had comparable spoken expressive language to Cluster A children, but with such small numbers this remains unclear. The children in Cluster A also had higher receptive vocabularies than children in Cluster C (*p* = .0007) and Cluster D (*p* = .0008), but not statistically different to the three children in Cluster B (*p* = .16).It is important to note that some children in this cluster reached ceiling level on the CDI and all scored highly. This measure, intended for children at early stages of linguistic development, is not sensitive enough to detect variation within this subgroup.

By contrast, their scores from the PLS-4 vary widely. Both the Expressive Communication and the Auditory Comprehension scores range from 50 (floor) to scores within the normal range. The PLS-4 Auditory Comprehension score did not reach significance on the Kruskal Wallis test meaning, for the 16 children able to complete this assessment, subgroups did not differ significantly on this score and a Dunn’s test was not completed.

Cluster A children presented with the strongest speech abilities of any cluster. Pairwise comparisons with Dunn’s tests indicated that the children in Cluster A had higher scores on all speech variables compared to Cluster B and Cluster C children. While Cluster A and B children had comparable age and receptive vocabularies, they had significantly different spoken language and speech abilities. The children in this Cluster A had the largest consonant repertoires, with all children using at least 19 consonants and some using the complete 24 consonants. The children in Cluster A scored significantly higher than the other three clusters on consonant accuracy. Children in Cluster A and D did not differ on use of Early 8 consonants (*p* = .28), use of CVC (*p* = .39), VC (*p* = .47), CCVC (*p* = .45) and CVCC (*p* = .12) syllables, or vowel accuracy scores (*p* = .057). Children in Cluster A were not routinely using alternative or augmentative communication (AAC), although some children did use visual schedules for routines.

### Cluster B: High Receptive, Low Expressive, High Gestures, Low Speech

Children in Cluster B did not differ on the Dunn’s test to children in Cluster A on measures of age (*p* = .3), receptive vocabulary (*p* = .16) and use of gestures (*p* = .2). Their mean age of 70.7 (SD 18.6) months was significantly older than the children in Clusters C and D. Two children in Cluster B had available scores from thee GMDS-ER. The mean NVDQ was 62.2 (*SD* 19.8, range 48.2–76.3). The three children in Cluster B differed from Cluster A on all speech variables. Cluster B children’s speech and spoken expressive vocabularies were similar to children in Cluster C. Pairwise comparisons indicate that children in Cluster D differed from Cluster B children on age (*p* = .011), number of Early 8 consonants (*p* = .011) and use of post-vocalic consonants (CVC: *p* = .011; VC: *p* = .009). All three children in Cluster B supplemented their limited verbal communication with AAC, including the use of spoken output devices (e.g., iPads), picture exchange systems, visual schedules, and key word signs.

### Cluster C: Low Language, Low Gestures, Low Speech

The three children in Cluster C had the lowest levels of language, nonverbal communication and speech capacity of any cluster. The children in Cluster C had a mean age of 44 (*SD* 4.4) months, younger than Clusters A and B. All three Cluster C children had available scores on the GMDS-ER. Mean NVDQ was 36.8 (*SD* 13.4, range 21.3–45.2). Cluster C children could be described as prelinguistic, all producing less than 6 recognizable words. Children in this cluster were unable to complete the PLS-4, and speech accuracy scores were unable to be calculated. Dunn’s comparisons indicate that children in Cluster C differed from Cluster A children on age and all communication measures. Their speech and spoken expressive vocabularies did not statistically differ to Cluster B children, although their receptive vocabularies (*p* = .041) and use of gestures (*p* = .039) were lower. Children in Clusters C and D did not differ on age, receptive and spoken vocabularies, or use of gestures. Their speech skills did differ, however, with children in Cluster D using more Early 8 consonants (*p* = .004) and post-vocalic consonants (CVC: *p* = 0.004; VC: *p* = 0.009). Although low-technology AAC methods (e.g., photographs for picture exchange, visual schedules, key word signs) were used with all three children in Cluster C, none of the children were consistently using AAC for functional communication.

### Cluster D: Low Language, Low Gestures, Developing Speech

The five children in Cluster D had a mean age of 45.2 (*SD* 7.2) months. Four children had available results from the GMDS-ER. The mean NVDQ was 63.6 (*SD* 30.1, range 45.8–108.3). The children in Cluster D were comparable to Cluster C children on age and although their receptive and spoken expressive vocabularies were larger than the Cluster C children, these differences did not reach significance on pairwise comparison using Dunn’s test. Interestingly, the two children in Cluster D who were able to complete the PLS-4 scored similarly to Cluster A children on Auditory Comprehension and Expressive Communication. Cluster D children differed from Cluster B and C on many speech variables, such as number of Early 8 consonants and use of post-vocalic consonants. Their consonant accuracy was higher than the Cluster B children but lower than Cluster A. The accuracy of vowel production was similar to Cluster A children (*p* = .057). Three of the five children in Cluster D occasionally used low-technology AAC, such as visual schedules or social stories, at times throughout their day. None of the children consistently used AAC for functional communication.

### Changes in Speech Capacity

The exact change across communication variables for each participant is reported in Table 5. The 11 participants in Cluster A from Time 2 includes all ten children from the Time 1 Cluster A and participant 5. Participant 5 is the last child to merge with this cluster, as depicted by fusion at a higher level on the Time 2 dendrogram. Participant 5 was included in Cluster C at Time 1, a cluster with low language and low speech ability. Cluster B remained stable from Time 1 to Time 2. This cluster includes 3 participants. Cluster C from Time 1 splits in two and forms Time 2 Cluster C (n = 3) and Cluster D (n = 5).

**Table 5:** Participants’ Time 2 results on communication measures and exact change over 12 months

Variable	Participants																					
	1	3	4	5	6	7	8	9	11	12	13	14	15	16	17	18	19	20	21	22	23	24
T2 Age	46	86	42	46	67	37	50	68	47	76	50	42	76	67	55	75	50	75	55	54	39	80
T2 CDI raw scores																						
RV	109	393	60	328	396	204	207	396	13	289	316	244	396	393	132	373	386	396	370	368	160	377
Change	78	30	59	48	1	203	99	0	7	0	82	84	0	4	121	14	42	34	14	16	92	6
EV	0	73	35	230	396	191	161	396	3	6	19	70	396	383	58	359	383	392	367	364	6	330
Change	0	73	35	100	1	190	90	0	2	2	14	48	0	22	58	6	50	35	21	12	− 16	13
Gestures	29	55	37	55	63	48	14	63	22	40	52	52	57	54	35	45	51	46	60	59	38	47
Change	7	13	14	12	0	32	12	0	14	0	− 2	10	3	5	29	3	9	0	5	2	8	1
T2 Independent analyses																						
Consonants	1	8	13	20	21	13	21	22	6	8	6	14	24	22	16	19	19	22	22	22	3	22
Change	− 1	3	3	6	3	7	5	1	2	4	0	4	1	3	4	1	3	1	4	3	− 5	*4*
Early 8	0	3	8	8	8	7	8	8	5	7	4	8	8	8	8	8	8	8	8	8	3	8
Middle	1	3	3	7	8	3	7	8	0	1	1	4	8	8	5	7	7	8	8	8	0	8
Late 8	0	2	2	5	5	3	6	6	1	0	1	2	8	6	3	4	4	6	6	6	0	6
Syllable shape																						
V	43.2	61.5	10.9	7.9	3.7	4.7	5.6	4.2	57.8	14.6	72.5	11.6	4.3	4.3	15.8	4.7	2.9	4.3	4.9	4.9	86.5	7.6
CV	56.8	33.8	67.4	58.9	59.9	46.7	46.1	62	41.4	75.6	27.5	72.5	56.4	60.7	31.6	61.7	22.9	60.9	60.5	61.1	13.5	61.8
CVC	0	4.6	17.4	21.2	21	29.7	33.7	18.7	0.9	9.8	0	10.1	19.6	19	34.2	20.1	40	19.3	20.4	19.8	0	17.2
VC	0	0	4.3	2	3.1	4.7	2.2	3	0	0	0	2.9	5.5	5.5	15.8	4	5.7	3.7	4.3	3.1	0	3.2
CCV	0	0	0	5.3	8.6	4.7	3.4	7.8	0	0	0	2.9	9.8	6.1	0	6	0	7.5	6.2	6.2	0	6.4
CCVC	0	0	0	0.7	1.2	6.3	3.4	1.8	0	0	0	0	1.8	1.8	2.6	0.7	11.4	1.9	1.2	2.5	0	1.3
CVCC	0	0	0	2.6	2.5	3.1	3.4	2.4	0	0	0	0	2.5	2.5	0	2.7	14.3	2.5	2.5	2.5	0	2.5
CCVCC	0	0	0	0	0	0	1.1	0	0	0	0	0	0	0	0	0	2.9	0	0	0	0	0
CVCCC	0	0	0	0	0	0	1.1	0	0	0	0	0	0	0	0	0	0	0	0	0	0	0
T2 Relational speech analyses																						
PCC		17.6	42.9	71.4	88.4	55.9	84.2	92.7		19.7	2.7	44.5	93.7	86.8	46.8	72.1	91.3	95.3	85.6	81.1		75.6
change		12.7	−	14.3	6.7	-	36.1	13		1	− 4.3	14.9	1	10	-	− 2.3	27	3.8	13.5	22.6		*12.1*
PVC		18.9	85.4	88.2	95.1	88.1	100	99.4		25.6	22.5	68.7	95.8	97	69.8	89.1	94.3	98.8	96.9	92.7		91.5
Change		8.4	−	6.1	4.1	−	10.6	2.7		0.6	− 13	0.1	− 4.2	4.5	-	1	2.9	0.3	− 0.3	7.6		*6.4*
PPC		18.2	59.2	78.6	91.3	67.6	90.2	95.6		21.9	9.7	53.2	94.6	91.4	54.9	79.3	92.3	96.8	90.5	86		82.4
Change		10.8	−	12.8	5.4	-	26.1	8.2		0.6	− 8.4	6.4	− 1.4	7.5	-	− 1.2	28.5	2.2	9.1	15.5		*9.4*

*T2* time two, 12 months after T1; Age in months, *RV* receptive vocabulary taken from CDI at T2, *EV* expressive vocabulary taken from CDI at T2, *change* change between T1 and T2 (T2-T1); Consonants is number of consonants in phoneme repertoire; Early Middle, Late 8 (Shriberg, 1993); *syllable shape* Proportion of each syllable type, *V* vowel, *C* consonant, *PCC* percent consonants correct, *PVC* percent vowels correct, *PPC* percent phonemes correct

### Communication Profiles and Trajectories of Development

**Fig. 3:**
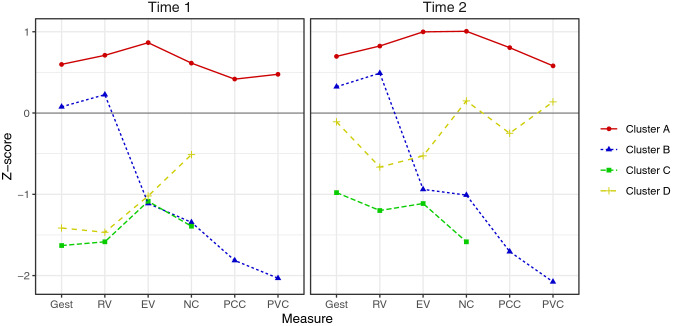
**a** Communication profiles of the Time 1 clusters. **b** Communication profiles of the Time 2 clusters

The communication profiles of the four clusters were plotted based on Time 1 (Fig. 3a) and Time 2 (Fig. 3b) scores for the six clustering variables. Differences in the Time 1 and Time 2 plots provides information regarding change in abilities and highlights possible predictor variables that may explain why some children in the cohort developed speech along a different trajectory to others.

### Cluster A

Cluster A children scored above the mean for the cohort on all communication variables at Time 1 and Time 2. Small improvements in their spoken expressive vocabularies, consonant repertoires and consonant accuracy can be seen across 12 months. It is important to note, that these children were at or close to ceiling levels on the CDI and consonant repertoire measures. One participant (participant 5) moved from Cluster C (low verbal, low gestures, low speech) at Time 1 to Cluster A (high language, high gestures, high speech) at Time 2. A plot of his individual change demonstrates a large improvement across all communication variables (Supplementary Material). Participant 5’s spoken expressive vocabulary, gestures, consonant repertoire, and PCC went from below mean compared to the cohort at Time 1 to above mean at Time 2.

### Cluster B

The three children in Cluster B presented with a unique communication profile characterized by high gestures and receptive vocabulary, with very low spoken expressive vocabulary and speech capacity. This profile remained consistent over time. There was very limited improvement in the communication ability of Cluster B children over 12 months. Their vowel accuracy was lower at Time 2 compared to Time 1.

### Cluster C

Time 1 Cluster C children were described as prelinguistic or minimally verbal. Visually, at Time 1 the children who ended up in Cluster C and D at Time 2 appear similar on measures of gestures, receptive vocabulary, and spoken expressive vocabulary. The three children who remained in Cluster C at Time 2 used less consonants at Time 1 than the children in Cluster D at Time 2. Over the 12 months, the children in Cluster C improved slightly in their use of gestures and receptive vocabulary but had no change in their spoken expressive vocabularies and remained at the prelinguistic stage. On average, the children in Cluster C used less consonants at Time 2 compared to Time 1. The Time 1 dendrogram (Fig. 2a) shows two of the children who ended up in Cluster C at Time 2, participant 1 and 7, fused to form a cluster low in the dendrogram illustrating similarity at this time. Participant 23 was different and did not fuse to participant 1 and 7 until much higher in the Time 1 dendrogram. Looking at the individual communication profile of participant 23, this child improved their use of gestures and receptive vocabulary over 12 months but regressed in their spoken expressive vocabulary and use of consonants (Supplementary Material).

### Cluster D

The five children in Cluster D at Time 2 presented as prelinguistic or minimally verbal at Time 1. These children had comparable use of gestures, receptive and spoken expressive vocabularies but used more consonants than the Cluster C children at Time 1. Time 2 plot (Fig. 3b) shows the children in Cluster D had a very different trajectory of communication development over 12 months. The five children in Cluster D, those using more consonants at Time 1, improved significantly more than those in Cluster C. These children went from below mean to near mean on all communication measures. The children in Cluster D also became verbal in these 12 months, as can be inferred from the inclusion of consonant and vowel accuracy measures at Time 2. Once these children began using spoken words, their consonant and vowel accuracy was at mean compared to the cohort.

## Discussion

This is the first longitudinal study to describe the different trajectories of speech development for subgroups of autistic children. Subgroups were formed using the same set of detailed communication variables used in Time 1 clustering (Broome et al., 2021) so the stability of these subgroups over 12 months could be described. Results suggest varied trajectories of speech development particularly for the children with ‘low language and low speech’ at Time 1. Some children who presented with limited language and speech capacity at Time 1 improved across all communication variables over 12 months and were talking at Time 2. Other children in this subgroup remained nonverbal. A child’s consonant inventory at Time 1 may predict better speech outcomes.

### Stability of Speech Subgroups of Autistic Children

Previous research (Broome et al., 2021; Chenausky et al., 2019; Kjelgaard & Tager-Flusberg, 2001; Rapin et al., 2009) has described subgroups of autistic children with specific speech profiles but, until now, no study has investigated the stability of these subgroups over time. While cross-sectional data provides a snapshot of a child’s speech capacity, longitudinal data is needed to inform of the likely speech outcomes for autistic children. Results of this study suggest membership of two subgroups from Time 1 remain stable, but the children in the third subgroup have varied outcomes.

The 10 children in Cluster A at Time 1 all remained in Cluster A at Time 2. These children presented with relatively high language, use of gestures and speech capacity. Overall, as the children in this subgroup were at or near ceiling on many of the communication variables in this study, minimal improvements were recorded. There was slight improvement in their consonant inventories and PCC scores over 12 months. These children are possibly indicative of the ‘average’ speech subgroup previously identified (Chenausky et al., 2019; Kjelgaard & Tager-Flusberg, 2001; Rapin et al., 2009) and may indeed follow the speech trajectory of neurotypical children already outlined in the literature. Past research reports high rates of mild articulation errors in older highly verbal autistic children (Cleland et al., 2010; Shriberg et al., 2001, 2011). This study does not explore the presence or absence of mild articulation errors. It is possible that some children in Cluster A present with mild speech errors, which would be interesting to investigate further in future research.

The three children in Cluster B at Time 1 remained in Cluster B at Time 2. Cluster B children presented with a unique communication profile of high receptive vocabulary and use of gestures, but low speech and low spoken expressive vocabulary. The communication ability of the three children in this subgroup showed very little improvement on the variables measured in this study and the unique communication profile remained constant over 12 months. The etiology of their speech and spoken expressive vocabulary difficulties needs to be explored further. Additionally, the possible interaction between predictor variables and spoken communication outcomes for these children is an interesting area for future research. All three children used effective augmentative and alternative communication (i.e. picture exchange, verbal output communication devices such as iPads, key word signs) suggesting their lack of verbal communication was unrelated to low communicative intent, a sentiment echoed by Saul and Norbury (2020). It is likely that Cluster B children present with a co-occurring SSD, characterized by a severely limited consonant repertoire and very low consonant and vowel accuracy. Differentially diagnosing a speech sound disorder in minimally verbal children is challenging (Chenausky et al., 2019; Strand et al., 2013). This study expands on the previous descriptive study of the speech capacity of these children (Broome et al., 2021) and adds valuable information regarding the trajectory of their speech development. Further information regarding imitated verse spontaneous speech, sequencing of speech sounds, vowel repertoire, and consistency of production would add vital information needed to differentiate the specific speech sound disorder in this subgroup.

The nine children with available longitudinal data from Time 1 Cluster C (low language, low speech) had three different communication trajectories. This result suggests that, particularly for children at very low levels of speech and language development, there is a need to be cautious when predicting spoken communication outcomes. While two children may appear very similar at a given point in time, they could have vastly different trajectories of speech development. In the current study, all nine children were described as having low language and low speech at Time 1. After 12 months, one participant moved to the high speech, high language subgroup (Cluster A). At Time 1, participant 5 had difficulty sitting and engaging in the language and single-word naming tasks. It is possible that his scores at Time 1 may not have reflected his underlying capacity. At Time 2, participant 5 was able to maintain attention and interest and completed all tasks presented.

The eight remaining children split to form two clusters at Time 2, Cluster C and D, with vastly different spoken communication outcomes. Age did not appear to predict communication trajectories for these children, as children in Clusters C and D did not differ on age. Children in Cluster C and D at Time 2 differed only in the number of consonants in their sound repertoire at Time 1. This preliminary result adds support to prior research reporting consonant inventories as one of the strongest predictors of spoken expressive language development in autistic children (Saul & Norbury, 2020; Wetherby et al., 2007; Yoder et al., 2015). The three participants with few consonants at Time 1 did in fact remain minimally verbal at Time 2 (Cluster C), with expressive vocabularies of fewer than six words and low speech capacity. The five children, originally with low language and low speech but more consonants, formed Cluster D at Time 2. These children presented with the most communication growth of any subgroup. The 12-month trajectory of this subgroup, from minimally verbal to verbal, offers valuable information and hope to parents, clinicians and researchers. The results suggest there may be the potential to identify the children likely to have better spoken communication outcomes. This is an important group to explore further.

### Possible Predictor Variables for Speech Outcomes

As previously described, a child’s early consonant inventory may predict their later spoken expressive vocabulary development. The results of this study also suggest that a child’s early consonant inventory may predict speech development more generally. This is an interesting avenue for future research investigating the trajectory of speech development with large cohorts of autistic children.

In this study, no single communication variable could differentiate the four subgroups at Time 1. For example, Cluster B children and Time 2 Cluster C children had comparable consonant inventories at Time 1. Instead, a combination of a child’s receptive vocabulary and their consonant inventory has the potential to describe their communication profile. These preliminary descriptive results may guide future research aiming to predict speech outcomes for autistic children. Large population studies of the speech of autistic children are required to reach conclusions regarding prevalence, causality, and outcomes.

### Communication Regression

An unexpected finding in this study was communication regression. Regression in autistic children has been reported in the literature for decades (Lord et al., 2004; Ozonoff et al., 2011, Shumway et al., 2011). Language is reported to be most frequently affected, encompassing loss of babbling, words, and word combinations (Barger et al., 2013; Borterberg et al., 2019). In a meta-analytic review of the literature, the mean age of regression in autistic children was reported to be 21.4 months (Barger et al., 2013). In this study, participants were at least 2;0 years and so regression was not expected. For some children, regression of speech capacity may in fact reflect their attempts at more complex word forms (i.e., consonant blends, polysyllabic words) rather than a true regression of skill. This would be reflected in reduced consonant and vowel accuracy, rather than a regression in consonant inventory or expressive vocabulary. This is likely true for the Cluster A children who regressed in phoneme accuracy (participants 13, 15, 18 and 21). One participant in this study regressed in speech and expressive language between 27 months (Time 1 age) and 39 months (Time 2 age). Although older than the average age for language regression reported by Barger et al. (2013), this age falls within the 6–36 month range provided by Luyster et al. (2005). Few prospective longitudinal studies of autistic children have captured regression (Borterberg et al., 2019) and this unexpected finding adds interesting information to this body of research.

### Limitations

Consideration should be given to the limitations of this study when interpreting the findings. Firstly, the small sample size in this study limited the statistical approach that could be applied to the longitudinal data. As each subgroup had a small number of children, communication profiles and trajectories were represented visually. The small sample size in each cluster limits our ability to generalize the findings of this study. It is important to remain cautious when interpreting the results and to see this study as a first step. Expanding this preliminary data with larger cohorts of children is important.

Secondly, a number of children in Cluster A reached near or at ceiling on the CDI. As a result, growth in receptive and expressive vocabulary and use of gestures was not able to be ascertained for these children using this measure. It is possible that more subgroups exist within the high language, high speech subgroup. Larger studies using more sensitive assessment measures for children at this level of functioning are needed to explore this possibility and describe this cluster in more detail.

Finally, this study recruited a heterogeneous cohort of children. The variation in age, level of cognition and functioning, and communication capacity made it difficult to select assessment tools appropriate for the whole cohort. Some participants were unable to complete the standardized language assessment (PLS-4), limiting the use of this measure in clustering. While spontaneous speech samples were collected for every participant, using speech samples for longitudinal comparison is not without limitation. Samples differed in the number of utterances and phonological complexity of words and some children used a large proportion of learnt words and phrases. For other children, it was challenging to ascertain the target word in their samples due to low speech accuracy. The variations between samples makes it difficult to draw comparisons between samples within the cohort (Stoel-Gammon & Williams, 2013) and to compare samples from the same child over time. For these reasons, speech data was based on single-word naming tasks in this study. Different single-word naming measures were required to capture each participant’s optimal speech capacity. For some highly verbal children in this study, a polysyllabic-word task could be completed. For less verbal children, a naming task using photographs of common objects was required and for children who were not yet verbal, speech data was taken from vocalizations produced during the assessment. Given these inconsistencies, it is important to interpret the results in this study with caution. One of the challenges in studying a heterogeneous cohort, is selecting appropriate assessment tools to capture each child’s optimal ability. Standardized assessments appropriate for autistic children across ages and levels of functioning are needed to advance our understanding of the inherent heterogeneity in autism (Kasari et al., 2013; Plesa Skwerer et al., 2016).

Replicating the results of this study with more homogeneous cohorts using consistent assessment measures is important. Cohorts with narrower age ranges would add valuable data. Unfortunately, given the challenges recruiting participants, often a balance needs to be achieved between recruiting enough children and limiting the ages of the participants.

### Future Research Directions

This is the first prospective longitudinal study to detail the speech development of autistic children. It is hoped that this preliminary data paves the way for future research in this area. Large population studies are needed to explore speech trends, trajectories and outcomes for autistic children. It would be interesting to investigate if a child’s consonant inventory and receptive vocabulary does indeed predict speech outcomes in this population. Additionally, replicating these findings with more homogeneous groups of children is important.

Future research aiming to differentially diagnosing SSDs in autistic children is needed. It remains unknown if autistic children can complete a speech imitation task, such as the Dynamic evaluation of motor speech skill (DEMSS; Strand & McCauley, 2019). Further information regarding their imitated verses spontaneous speech, sequencing of speech sounds, vowel repertoires, consistency of productions and the utility of using echolalic speech in assessment is required to differentiate the specific speech sound disorder in this subgroup. Some autistic children with a co-occurring SSD may require targeted speech intervention. Some researchers have begun to investigate the outcomes of speech-based intervention with these children (Beiting & Maas, 2020; Chenausky et al., 2018) and this is an important avenue for further research.

### Clinical Implications

Clinicians are frequently asked by parents of autistic children, particularly those who are less verbal, of the probable prognosis of their child’s communication. Parents want to know if their children will ever talk. While there is some literature to guide clinicians regarding the possible language outcomes (see Brignell et al., 2018 for review), there is a large gap in the literature to inform of the expected prognosis of autistic children with a suspected co-occurring SSD. Until the body of literature detailing the trajectories of speech development grows, clinicians will be unable to provide parents with an informed response. This study suggests that for some autistic children with a suspected SSD, very little development in speech or expressive language occurs over 12 months. Given this limited progress, clinicians should consider targeted speech interventions for these children. Some minimally verbal autistic children can make significant improvements in their speech and expressive language in 12 months and it appears that their early consonant inventory and receptive vocabulary may be important predictors to this growth. Clinicians should be assessing the consonant inventory and receptive language capacity of children and developing a child’s capacity in these areas through intervention.

## Supplementary Information

Below is the link to the electronic supplementary material.

Supplementary Figure 1. Participant 5 trajectory of communication development (PNG 253 kb)

Supplementary Figure 2. Participant 23 trajectory of communication development (PNG 226 kb)
