# Supplementary figures and images for: Speech Development Across Subgroups of Autistic Children: A Longitudinal Study
Source: J Autism Dev Disord. 2022 Apr 19;53(7):2570–86. doi: 10.1007/s10803-022-05561-8 (PMC10290604; doi:10.1007/s10803-022-05561-8)

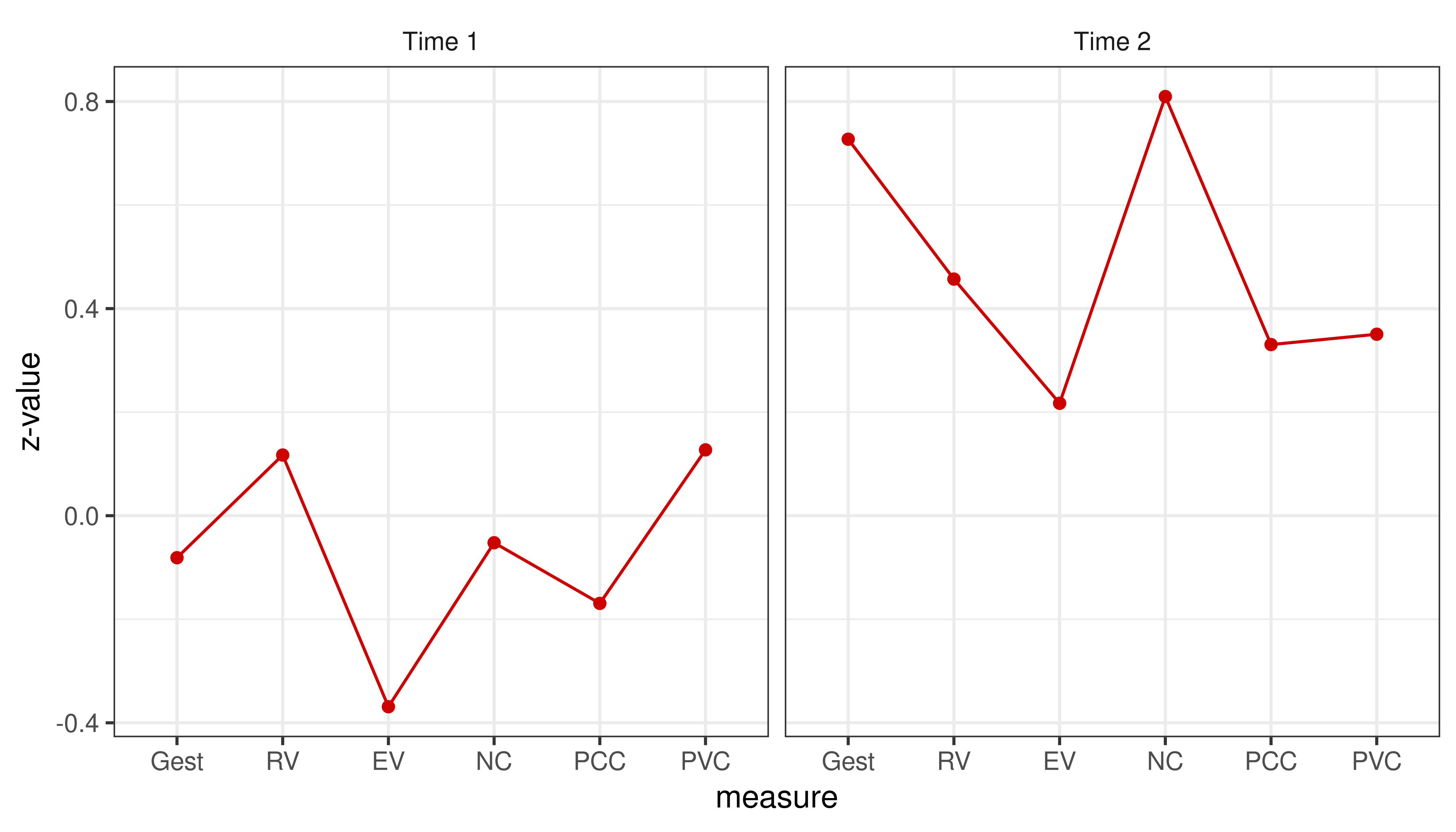

Supplement: Supplementary file 1 — Supplementary Figure 1. Participant 5 trajectory of communication development (PNG 253 kb) [file 10803_2022_5561_MOESM1_ESM.png]

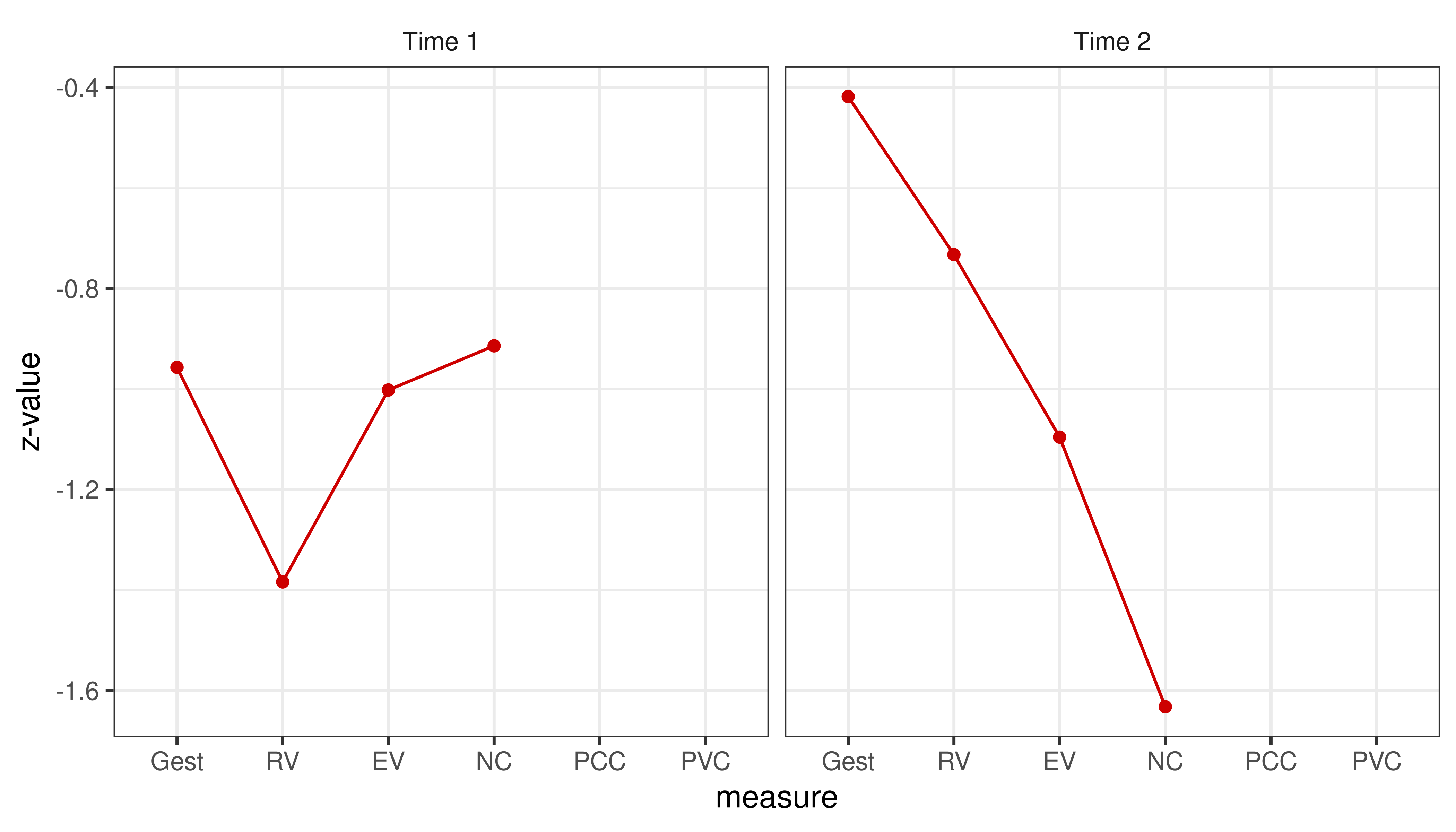

Supplement: Supplementary file 2 — Supplementary Figure 2. Participant 23 trajectory of communication development (PNG 226 kb) [file 10803_2022_5561_MOESM2_ESM.png]
